# Simultaneous bilateral reintervention using one-step endoscopic ultrasound-guided biliary drainage for severe acute cholangitis caused by malignant hilar biliary obstruction

**DOI:** 10.1055/a-2325-2624

**Published:** 2024-06-05

**Authors:** Takeshi Ogura, Atsushi Okuda, Saori Ueno, Nobu Nishioka, Hiroki Nishikawa

**Affiliations:** 1 38588 Endoscopy Center, Osaka Medical and Pharmaceutical University Hospital, Takatsuki, Japan; 2 13010 2nd Department of Internal Medicine, Osaka Medical and Pharmaceutical University, Takatsuki, Japan

Endoscopic retrograde cholangiopancreatography (ERCP) is a standard technique for endoscopic biliary drainage in malignant hilar biliary obstruction (MHBO). In cases of unresectable MHBO, the deployment of bilateral self-expandable metal stents (SEMSs) may be indicated 1 2 . However, with the recent development of systemic chemotherapy 3 and local tumor treatment by endoscopic radiofrequency ablation, the frequency of reintervention for stent dysfunction may increase and reintervention after bilateral SEMS deployment may be challenging. If reintervention under ERCP guidance is needed, percutaneous transhepatic biliary drainage (PTBD) is considered. As with other biliary drainage techniques, endoscopic ultrasound (EUS)-guided hepaticogastrostomy (HGS) and hepaticoduodenostomy (HDS) can be considered bilateral reintervention techniques 4 5 .

If patients have severe acute cholangitis, both biliary drainage techniques are rapidly needed. Furthermore, in acute cholangitis, there can be leakage of infected bile after tract dilation. Therefore, a one-step technique such as stent deployment without tract dilation may be helpful in preventing adverse events. If bilateral drainage is needed, the one-step technique is also preferable from the perspective of shorter procedure time. Recently, a novel partially covered SEMS with a fine-gauge stent delivery system (7 Fr; BileRush Advance, Piolax Medical, Kanagawa, Japan) has become available. This stent might be useful for one-step EUS-guided biliary drainage (EUS-BD). A case of simultaneous bilateral reintervention using one-step EUS-HGS and EUS-HDS for severe acute cholangitis due to MHBO is described.

An 89-year-old man underwent bilateral SEMS deployment for unresectable MHBO, and subsequently underwent several reinterventions for stent dysfunction. The patient was admitted with severe acute cholangitis caused by stent obstruction. Because of previous failed reintervention under ERCP guidance, EUS-BD was attempted. If EUS-HGS had been performed first, stent dislocation could have occurred during scope insertion into the duodenum and therefore EUS-HDS was attempted first. When detecting the right hepatic bile duct, it is important to prevent duodenal perforation, so the scope position was adjusted using fluoroscopic guidance. The posterior bile duct was punctured using a 19G needle and contrast medium was injected ( Fig. 1 **a** ). A 0.025-inch guidewire was deployed ( Fig. 1 **b** ). Insertion of the stent delivery system was attempted without tract dilation, and the stent was successfully deployed from the posterior bile duct to the stomach ( Fig. 1 **c** ). The echoendoscope was then pulled back into the stomach and the left intrahepatic bile duct was identified. After bile duct puncture had been performed with a 19G needle, cholangiography was performed with injection of contrast medium ( Fig. 1 **d** ). After guidewire deployment, stent deployment from the intrahepatic bile duct to the stomach was successfully performed without tract dilation ( Fig. 1 **e** , Video 1 ). The patient’s cholangitis was completely resolved by these drainage procedures without any adverse events being noted.

**Fig. 1:**
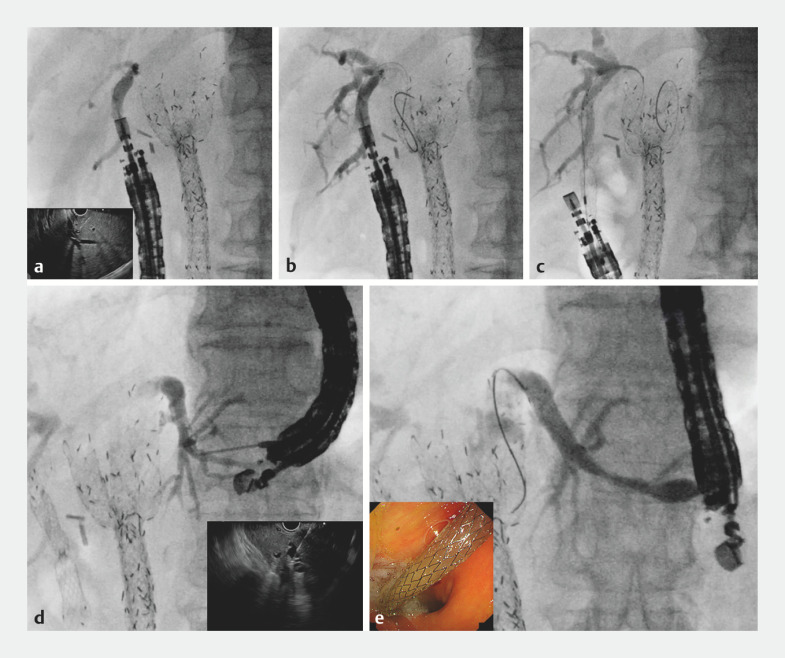
Fluoroscopic images showing: **a** injection of contrast medium after puncture of the posterior bile duct using a 19G needle (inset, endoscopic ultrasound [EUS] image); **b** deployment of a 0.025-inch guidewire; **c** successful deployment of a novel metal stent with a fine-gauge stent delivery system without tract dilation; **d** injection of contrast medium after puncture of the left intrahepatic bile duct using a 19G needle (inset, EUS image); **e** successful deployment of a novel metal stent with a fine-gauge stent delivery system without tract dilation (inset, endoscopic appearance of the stent).

Simultaneous bilateral reintervention is performed using a one-step technique under endoscopic ultrasound guidance with placement of novel metal stents that have a fine-gauge stent delivery system.Video 1

In conclusion, simultaneous bilateral reintervention using one-step EUS-HDS and EUS-HGS may be feasible and safe for such patients.

Endoscopy_UCTN_Code_TTT_1AS_2AH

## Notes

Endoscopy E-Videos https://eref.thieme.de/e-videos
*E-Videos* is an open access online section of the journal *Endoscopy* , reporting on interesting cases and new techniques in gastroenterological endoscopy. All papers include a high-quality video and are published with a Creative Commons CC-BY license. Endoscopy E-Videos qualify for HINARI discounts and waivers and eligibility is automatically checked during the submission process. We grant 100% waivers to articles whose corresponding authors are based in Group A countries and 50% waivers to those who are based in Group B countries as classified by Research4Life (see: https://www.research4life.org/access/eligibility/ ). This section has its own submission website at https://mc.manuscriptcentral.com/e-videos .
